# Biocompatibility of Blank, Post-Processed and Coated 3D Printed Resin Structures with Electrogenic Cells

**DOI:** 10.3390/bios10110152

**Published:** 2020-10-22

**Authors:** Cacie Hart, Charles M. Didier, Frank Sommerhage, Swaminathan Rajaraman

**Affiliations:** 1 NanoScience Technology Center, University of Central Florida, 12424 Research Parkway, Suite 400, Orlando, FL 32826, USA; chart@knights.ucf.edu (C.H.); CharlesMDidier@knights.ucf.edu (C.M.D.); frank.sommerhage@ucf.edu (F.S.); 2 Department of Materials Science & Engineering, University of Central Florida, 12760 Pegasus Dr., Orlando, FL 32816, USA; 3 Burnett School of Biomedical Science, University of Central Florida, 6900 Lake Nona Blvd, Orlando, FL 32827, USA; 4 Department of Electrical & Computer Engineering, University of Central Florida, 4328 Scorpius St., Orlando, FL 32816, USA

**Keywords:** three-dimensional (3D) printing, HL-1 rat cardiomyocyte cells, biocompatibility, post-processing

## Abstract

The widespread adaptation of 3D printing in the microfluidic, bioelectronic, and Bio-MEMS communities has been stifled by the lack of investigation into the biocompatibility of commercially available printer resins. By introducing an in-depth post-printing treatment of these resins, their biocompatibility can be dramatically improved up to that of a standard cell culture vessel (99.99%). Additionally, encapsulating resins that are less biocompatible with materials that are common constituents in biosensors further enhances the biocompatibility of the material. This investigation provides a clear pathway toward developing fully functional and biocompatible 3D printed biosensor devices, especially for interfacing with electrogenic cells, utilizing benchtop-based microfabrication, and post-processing techniques.

## 1. Introduction

Conventional micro/nanofabrication techniques allow for the realization of micron- and nanoscale features; however, these techniques have several disadvantages, including expensive and time-consuming processes when altering the design of a device, difficulty in scaling up from prototyping to bulk manufacturing, and 3D geometric design requirements [1,2,3,4,5,6,7]. 3D printing, also known as additive manufacturing or rapid prototyping, has recently become an important technology for revolutionizing biosensor and microfluidic device development because of its ability to overcome most of the disadvantages associated with traditional microfabrication techniques [1,2,3,4,5,6,7,8]. Unlike traditional microfabrication techniques, 3D printing does not require the need for a cleanroom facility, thus allowing for simple implementation, low costs, and rapid fabrication [6].

3D printers generally define a three-dimensional object from a computer-aided design (CAD) model layer-by-layer in an additive process [5,7]. The most prevalent types of 3D printing utilize polymers, and these types are namely fused deposition modelling (FDM), micro-stereolithography (µSLA), and digital light processing (DLP). FDM printing involves the extrusion of small streams or beads of a material, such as polylactic acid (PLA), that immediately harden to form the structural layers of the final device [5,7]. FDM printing is the easiest to implement and the most cost-efficient of these three methods; however, limitations exist in the shapes and feature sizes that can be fabricated [5,7]. Both µSLA and DLP printing produce objects in a layer-by-layer fashion via the photopolymerization of a liquid resin. These technologies may also be referred to as vat photopolymerization or resin 3D printing [5,7,9]. Both µSLA and DLP printing can produce highly accurate, isotropic objects with micron-scale feature sizes and a smooth surface finish. The difference between these two types of printing lies in how the layers are cured. In µSLA printing, a laser beam and digital mirrors are used to photocure each portion of every single layer of the object, whereas in DLP printing, the entirety of each layer of the object is cured at once using a projected display [4,5,7,9,10]. With respect to the application of these types of 3D printed interfaces in biology, especially in the realm of in vitro studies, µSLA and DLP 3D printing lend supplemental benefits outside of the ease of microfabrication. Many cell types prefer micro/nano textured surfaces, which can be easily achieved by these methods. Such surfaces allow cells to better sense and respond to their environment [11,12,13]. It is important to note that the mechanical properties of such commercial resins are not suited for every application in the space of biomedical micro-electromechanical systems (BioMEMS) devices, and the choice of commercial resins can be motivated by a variety of factors, including: the ability to rapidly structure microsystems, the ability to access to ready-to-use materials and mechanically well-characterized materials, as well as cost, consistency, and ease of procurement. If other material properties are desired such as conformability, silcones and hydrogels remain as alterative 3D-printable materials potentially of interest [14,15,16].

3D printing allows for the accurate and rapid design and fabrication of monolithic devices. Although this technology has revolutionized MEMS and microfluidics fields, to our knowledge, little investigation into the biocompatibility of the most commonly used commercial 3D printing resins has been performed to date. This is especially true for electrogenic or electrically active cells (e.g., neurons, cardiomyocytes) where no biocompatibility reports have been published as far as our knowledge goes [1,2,8,9,10,17,18,19,20,21,22,23,24,25,26,27]. Many of these “off-the-shelf” 3D printing liquid resins can be purchased directly from the individual printer manufacturers, and as a result their compositions are proprietary. Thus, the constituent materials, specifically the photoinitiators, surfactants, solvents, and shelf-life extending compounds, are unknown. This lack of information is a barrier to wider adaptation of 3D printing in the bioelectronic, biosensor, and microfluidic communities, as material biocompatibility is of utmost concern for cells interfacing with these materials. Some groups have studied some of these resins with little luck in having any extended cell viability, except for the few groups that add prolonged UV treatment to the usual post-print processing [1,2,4,7,8,18,19,28,29]. Several of these materials are methacrylate-based as deduced from their materials safety data sheets (MSDS) [30,31,32,33,34]; however, their exact compositions remain proprietary. Studying these resins in cell culture and improving their biocompatibility through post-process treatments is of immense interest to the community and provides multiple advantages. First, the ability to design constructs for in vitro biological work solely based on 3D-printing microfabrication strategies means that novel and monolithic design approaches can be explored. Second, the application of post-processing treatments begins to reveal a larger concept of compound material biocompatibility, by posing the questions of the exact material changes occurring during each step. While some effects are readily known and the effects of certain treatments can be hypothesized, such approaches could allow for the identification of the exact components of each resin.

To test material biocompatibility, a cell type that has been well studied and has predictable growth patterns should be used. HL-1 cells are derived from rat atrial cardiac myocytes and are the only cardiomyocyte cell line currently available that can continuously divide, spontaneously contract, and maintain a differentiated adult cardiac phenotype [28,29]. Additionally, this cell line has been widely characterized using optical, electrophysiological, immunohistochemical, and pharmacological methods [28,29].

In this paper, we explore the feasibility of using resin 3D printing for in vitro biological applications by testing the biocompatibility of several commercial resins. By utilizing commercially available resins and standard BioMEMS coatings, we aim to demonstrate how several standard processes can be combined to provide enhanced biocompatibility, which in turn will create higher accessibility to 3D printed BioMEMS fabrication for the creation of novel, in vitro electrogenic assays. With respect to the selection of post-process treatments for 3D printed constructs, the treatments themselves should also be accessible to most labs. To that end, coatings that are known to enhance biocompatibility and are accessible to all labs, i.e., SU-8 [35,36], gold [36,37,38], polystyrene [39], Medco/polyethylene terephthalate (PET) [40], and polydimethylsiloxane (PDMS) [41] to enhance biocompatibility, were tested for each material. With respect to post-processing surface treatments, we examined isopropyl alcohol (IPA) treatment (a common solvent for cleaning freshly printed Methacrylate-based resin parts to remove the uncured monomer [42]); ultraviolet (UV) post-curing (a common step to improve 3D printed final structural properties due to enhanced crosslinking, as well as a common sterilization method prior to cell culture [9,43,44]); thermal baking (a common post-treatment for achieving final, post-print properties [9]), and autoclaving (a common sterilization technique [45] that may encourage further polymerization due to the high-pressure environment [46,47,48]). Additionally, several resins were tested, as each offers varying mechanical and optical properties, which lends themselves to be useful in multiple applications. Indirect and direct characterization of the biocompatibility of these resins was subsequently thoroughly characterized.

## 2. Materials and Methods

### 2.1. 3D Printed Resin Chips

**Figure 1:**
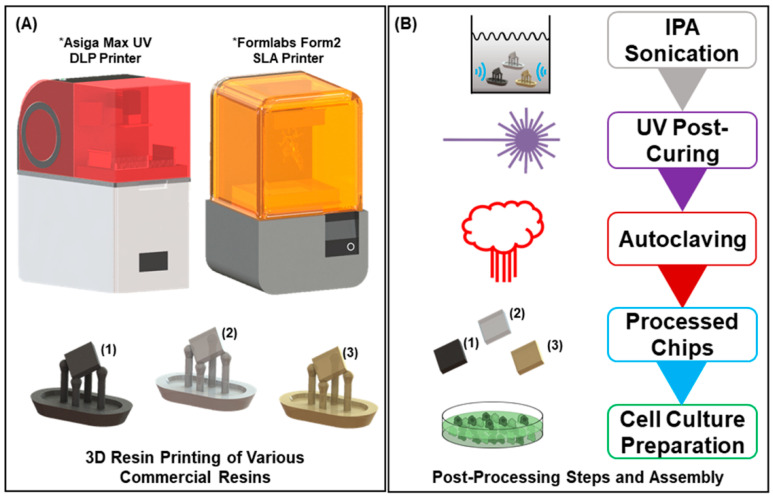
Resin chip fabrication: (**A**) Schematic of the Asiga Max UV digital light processing (DLP) 3D printer and the Form2 stereolithography (SLA) 3D printer and associated printed resin chips (Flexible Resin in black, Clear Resin/High Temp Resin in grey, and Dental Resin in brown). The printer can cure many different commercial methacrylate-based photopolymer resins, allowing for a variety of sampling materials, provided the photoinitiator absorbs between 385 and 405 nm. Each chip comes printed on a raft and support structure (as shown in the schematic) and must be singulated before post-processing. (**B**) Resin chip post-processing stages. Each of the indicated steps as well as the order was the final sequence used for a full regiment of resin treatment. After full post-processing, the chips can then be assembled in 48-well plates and sterilized for cell culture.

Computer-aided design of the sample chips (4 mm × 4 mm × 1 mm) was performed in SolidWorks (Dassault Systems, Velizy-Villacoublay, France). The chips were subsequently sliced and printed (Figure 1A) using either a combination of PreForm and a desktop Form2 µSLA 3D printer (both from FormLabs, Somerville, MA, USA) or Composer and an Asiga MAX UV27 DLP 3D printer (both from Asiga, Sydney, Australia) depending on the resin type used. The Form2 uses a 405 nm laser with a 140 µm spot size for curing, while the Asiga uses a 385 nm light emmiting diode (LED) with a pixel size of 27 µm.

The resins tested for biocompatibility were Clear Resin (FLGPCL04), Flexible Resin (FLFLGR02), Dental LT Clear Resin (DLFLCL01), and High Temp Resin (FLHTAM01) from FormLabs (Somerville, MA, USA) and the GR-10 Clear from Pro3dure (Iserlohn, Germany). All resins tested were printed on the Asiga printer, except for Flexible Resin, which was printed on the Form2.

After printing, the 3D-printed chips were subjected to numerous treatments to increase cell viability (Figure 1B). This figure demonstrates the order in which the treatments were performed. The post-processing treatment steps ranged from no treatment to a full treatment comprising all the different steps, but they were always performed sequentially. The full regimen of the post-processing treatments that could be combined is as follows, and the detailed methods are listed as the following: sonication rinse in fresh 70% isopropyl alcohol (IPA), ultraviolet light (UV) post-print curing, dry thermal post-baking at 80 °C, and full autoclaving cycle at 135 °C. The individual variations were performed solely for the FormLabs Clear Resin, to rapidly and efficiently idenitfy the major changes in efficacy of post-process treatments to biocompatibility, which were then to be applied to all resins.

IPA-treated chips were washed and sonicated at 40 kHz in fresh 70% IPA (Cole Parmer, Vernon Hills, IL, USA) for 15 min in a sonication bath (Fisher Scientific, Hampton, NH, USA). UV-cured chips were cured for 6 min at 60 °C in the Form Cure UV post-curing station (FormLabs, Somerville, MA, USA), which operates at a wavelength of 405 nm. The extended UV treated chip was cured for an additional 30 min in the same curing station. Thermally treated chips (not shown in Figure 1B) were baked in a benchtop oven (Fisher Scientific, Hampton, MA, USA) at 80 °C for 1 h. Autoclave treated chips were affixed to glass slides (Fisher Scientific, Hampton, NH, USA) using biocompatible Kapton^®^ polyimide tape (Dupont, Wilmington, DE, USA) and autoclaved in a SterilElite16™ autoclave (Fisher Scientific, Hampton, NH, USA) for a two-hour cycle, which comprised one-hour heating followed by an one-hour drying cycle at 135 °C. Since autoclaving includes a thermal step, resin thermal treatment is not depicted in Figure 1B. As a measure between the negative controls (listed in the Negative and Positive Controls section) and the post-treated resin samples described in the section (above), we also prepared resin chips that were not cleaned or treated in any way after removal from the 3D printers, aside from gently wiping away of any excess uncured resin.

After the post-printing treatments, some of the chips were coated with materials that are prevalent in BioMEMS and microfluidic devices. These coatings included 10 µm thick polydimethylsiloxane (PDMS) (Sylgard-184, Dow Corning, Midland, MI, USA) mixed in the standard 10:1 polymer to curing agent ratio; 5 µm thick polystyrene (PS, Millipore Sigma, St. Louis, MO, USA) mixed into tetrahydrofuran (THF) (Fisher Scientific, Hampton, NH, USA) to create a 10% w/v solution; 20 µm thick SU-8 (Gersteltec, Pully, Switzerland); 100 µm thick biolaminate layer consisting of a polyethylene terephthalate (PET) film with a single side coated with a Medco adhesive (Medco/PET) (Medco Labs, Bedford Heights, OH, USA); and 75 nm thick gold (Ted Pella, Redding, CA, USA). Chips with PDMS, PS, and SU-8 were dip-coated and cured at 60 °C for 1 h. Gold coated chips were sputter coated in a Quorum Q150T Sputter Coater (Quorum Technologies, Lewes, UK), and the biolaminate sheet was cut to size and applied to the chips, with the Medco adhesive acting as the bonding layer. All thickness measurements were performed using a scanning electron microscope (SEM) (JEOL, JSM-6480, Tokyo, Japan). FormLabs Clear Resin chips were coated with each of the afforementioned materials to evaluate the effect on biocompatibility as stated earlier, while each of the other resins were only coated with PDMS.

**Figure 2:**
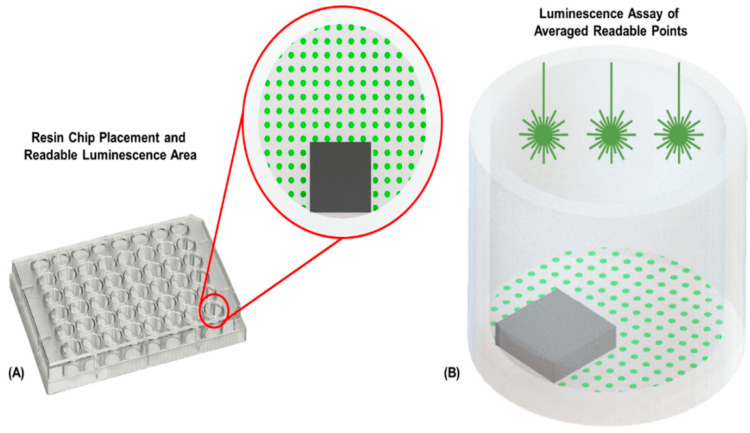
Luminescence assay. (**A**) Image of a 48-well plate with schematic close-up demonstrating the placement and relative size of the resin chips in the wells. The grid of green dots represents the sampling points of the luminescence assay. (**B**) Schematic view of the luminescence assay process, illustrating the averaged luminescence leading to an output of percent viability. The stars illustrate the source light hitting the surface of the liquid in the culture well.

The fabricated and post-processed chips were subsequently adhered to a sterile 48-well cell culture plate (Fisher Scientific, Hampton, NH, USA) with biocompatible 353ND epoxy [49] (Epotek, Billerica, MA, USA) mixed at a 50:1 epoxy to curing agent ratio for increased transparency. The chips were placed in one side of the well (Figure 2A) in order to expose sufficient area for the plate reader analysis. The plate was subsequently placed into an oven overnight at 45 °C to allow the epoxy to cure. For each experimental set, *n* = 6 samples were prepared.

The plates were then sterilized, first by washing each well two times with phosphate buffered saline (PBS) (Gibco, Waltham, MA, USA) for five minutes. As the next step, the wells were washed with 70% ethanol (Fisher Scientific, Hampton, NH, USA) for 10 min. The plate was subsequently UV sterilized in a biosafety cabinet (NuAire, Plymouth, MN, USA) for 30 min and then washed with sterile water four times for five minutes each.

### 2.2. HL-1 Cell Culture

The sterile 48-well plate fixed with the chips was first coated with a fibronectin/gelatin (both from Millipore Sigma, Burlington, MA, USA) extracellular matrix solution to allow for cell adhesion to the surface and then incubated at 37 °C with 5% CO_2_ overnight. The fibronectin/gelatin solution was aspirated just prior to cell plating and replaced with 500 µL of supplemented Claycomb media (Millipore Sigma, Burlington, MA, USA).

To culture the HL-1 cells, 10 mL of HL-1 wash medium (Millipore Sigma, Burlington, MA, USA) was first added to an empty 15 mL centrifuge tube and incubated in a 37 °C water bath. A cryogenic vial containing the cells was then removed from liquid nitrogen storage, and the cells were rapidly thawed in a 37 °C water bath for approximately two minutes. The contents of the cryogenic vial were subsequently transferred to the 15 mL centrifuge tube containing the prewarmed HL-1 wash medium. The tube was then centrifuged at 500× *g* for five minutes. After centrifuging, the wash medium was aspirated from the centrifuge tube, and the cell pellet was gently resuspended in 1 mL of supplemented Claycomb medium. Cells were then counted using a standard haemocytometer protocol with trypan blue (Gibco, Waltham, MA, USA). Typically, each cryogenic vial contains around 1.5 million cells. Since the optimal plating density for a 48-well plate is approximately 30,000 cells/well [25], approximately 20 µL of the cell suspension was added to each well. The exact amount depends on the cell density in a specific vial and is typically determined at this step in the process.

Culture plates were incubated at 37 °C with 5% CO_2_ and were allowed to reach confluency (3 days). Cell culture medium was exchanged daily. After the cells reached confluency, an ATP assay was performed to assess cell viability [50]. A Promega CellTiter-Glo (Promega, Madison, WI, USA) ATP assay was prepared according to its protocol [50] and is briefly described here: 200 µL of a supplemented Claycomb medium along with an equal amount of the ATP assay reagent was added to each well, mixed for two minutes to induce lysis, and then incubated at room temperature for ten minutes to stabilize it for the procurement of a luminescent signal. Luminescence was subsequently recorded for each well using a SpectraMax i3x microplate reader (Molecular Devices, San Jose, CA, USA) in a 48-point well scan mode (Figure 2A,B).

HL-1 cells were also optically observed using inverted microscopy (Nikon TS2, Nikon, Tokyo, Japan). Images of the cell growth over time and during beating were procured using a camera (Amscope, Irvine, CA, USA). Live/dead fluorescence images of the cells were taken using a Keyance BZ-X800 All-in-One Confocal Microscope (Keyance, Tokyo, Japan). These cells were first plated on standard microscope slide-sized (25 mm × 75 mm × 1 mm) treated resin pieces adhered to a glass microscope slide using 353ND epoxy to make the resin transparent at DIV05. An Invitrogen Live/Dead Cell Imaging Kit (Carlsbad, CA, USA) was used for fluorescent staining, where live cells fluoresce green and dead cells fluoresce red.

### 2.3. Negative and Positive Controls

In order to ensure the validity of the experimental protocol, both negative and positive controls were designed to clearly demonstrate uninhibited cell death and growth respectively. For the negative controls, 6 mL of each uncured resin was mixed with 24 mL of supplemented media to create a 1/4 (v/v) solution. The purpose of this solution was to “leach” the resin into the media (i.e., monomers, photoinitiators, solvents, etc., in their native form with toxic moieties [27,30,51]), so that potential cytotoxic compounds (which are normally removed during curing and post-processing steps) could be left in the media. Cells were plated as described in the previous section and adhered to the culture well surface (*n* = 6 for each resin), at which time point the “leached” media was used (moving forward) to supplement the cultures. For the positive control, cells were plated until adherent as above, and cultured for the duration of each experimental protocol without any resin (cured or uncured) present in the wells.

## 3. Results and Discussion

The positive and negative controls each serve to highlight the findings of the biocompatibility study described in the paper. Positive control wells were consistently 99.99% viable as expected. The negative control wells were nonviable (0% viability) within 12 h of the “leached” supplemented media being introduced in culture. This result held for all instances of every uncured resin leached media that was used. Uncured resin would most certainly never be used in the fabrication of a BioMEMS construct, as uncured resins contain shelf-life stabilizers, solvents, monomers, UV-blockers, and photoinitiators [27,30,51], and these are likely polymerized (or expelled) with the solvent evaporating out of the construct as the 3D structure is cured. Again, it is important to note that commercial resins are largely proprietary, so exact chemical compositions remain unknown and are an area of interest for future studies. These controls serve to demonstrate the necessity of the post-curing steps, including sufficient UV/thermal curing of the resin, which would ensure the removal of these overtly cytotoxic compounds [52,53] that may still be present in trace quantities after the initial structural curing of the resin during 3D printing.

The resins selected were based on both their commercial availability and recommended base resins (by the manufacturer) for the respective 3D printing platforms. These resins according to the manufacturers are the most used products in BioMEMS, microfluidics, dentistry, artistic endeavours, and other fields. As a starting basis for comparison, the various iterations of printed materials are deemed biocompatible with HL-1 cells if cell viability exceeded 85.00% [54]. This viability marker extends from both the ISO 10993-5 standard and the European Union Reference Library European Centre for Validation of Alternative Methods (EURL ECVAM) [54], as well as visual observations of cell growth and beating. The percentages shown are based on arbitrary luminescence units, corresponding directly to a culture’s population viability (average *n* = 6). Each experimental group was accompanied by a set of positive controls (no resin; *n* = 6; 99.99% average viability across experiments) that demonstrated the healthy viable nature of the cells used in a culture experiment. For each of the resins as mentioned previously, a negative control consisting of the uncured resin leached media (*n* = 6; 00.00% average viability) was performed.

**Figure 3:**
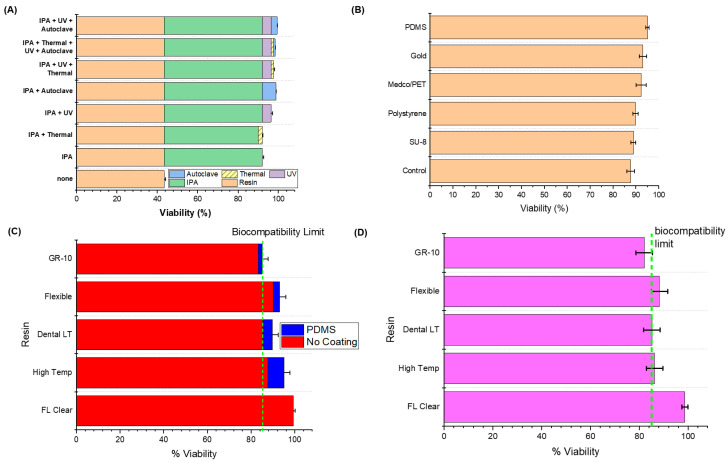
(**A**) Biocompatibility results of incremental improvements in biocompatibility during post-processing for FormLabs Clear V4: Clear Resin alone showed the lowest survival rate, while washing with isopropyl alcohol (IPA) resulted in the greatest increase of survival. Surprisingly, thermal treatment after IPA washing reduced the biocompatibility slightly even though the result was above the accepted values for HL1 cells. The combination of IPA, UV, and autoclave resulted in the greatest collective viability. (**B**) Coating encapsulation of FormLabs’ High Temp Resin to improve biocompatibility: All samples were washed with IPA, exposed to UV for 6 min, and subsequently autoclaved. SU-8 and polystyrene had a rather small impact on the biocompatibility. Medco/PET and gold resulted in slightly better biocompatibility, and encapsulation in PDMS provided the best results and was subsequently used. (**C**) Biocompatibility of the different resins before and after PDMS coating encapsulation. (**D**) Biocompatibility results of the different resins where the chips were only coated with fibronectin/gelatin solution as opposed to the entire culture well. Error bars here are standard error, and *p* < 0.01 for each of the four graphs.

Experimentally, the FormLabs Clear Resin [30] was tested first (Figure 3A), as it is the simplest and most widely available resin accessible for this type of work. All viability numbers mentioned in this section are an average of *n* = 6 samples. Untreated (printed; no wash or post-processing) FormLabs Clear Resin (FLGPCL04) was first tested as a basis of comparison and demonstrated a 43.39% ± 0.70% (mean ± standard deviation for all such numbers from now on) viability. We expect such a high cytotoxicity because of the presence of the various moities as hypothesized earlier [52,53]. Next, the post-processing treatments were tested in a compound manner, beginning with the sonication in fresh 70% IPA, a step routinely recommended to clean a resin-based 3D printed structure. The IPA wash alone resulted in a 91.99% ± 0.76% viability, a dramatic increase in biocompatibility for this resin. This dramatic increase in viability was expected as well, as the IPA rinse removes any uncured resin and other particles that remain on the structure after the printing process [42]. Next, an IPA and a UV post-cure was tested, resulting in a 96.35% ± 0.71% viability. The combination of the IPA rinse and UV curing likely results in an increase in viability due to the combination of the removal of uncured resin [42] and further crosslinking of the polymer in the UV curing station due to free radical initiated polymerization [9,43,44]. IPA and a thermal bake were then tested together resulting in 89.98% ± 0.39% viability, prompting a comparison with an IPA and autoclave regimen, which resulted in a viability of 98.71% ± 0.24%. From this result, we are able to hypothesize that while the addition of only thermal baking enhances the mechanical properties of the resin [9], no further crosslinking is achieved; however, the high pressure environment of an autoclave step may in fact further crosslink methacrylate-based resins, as previously mentioned in literature [46,47,48]. The clear benefits from combining IPA washing with UV post-curing and IPA with autoclaving prompted the final compound post-processing treatment regimen comprising IPA sonication, UV post-curing, and subsequently autoclaving, which resulted in the highest reported HL-1 cell culture viability for this type of resin of 99.31% ± 0.17%.

Although the combined treatments greatly increased the overall viability of the cultures, the Clear Resin itself suffered some minor visible damage from the heat and pressure inherent in an autoclave step. Due to this, the next resin tested was the FormLabs High Temp Resin (FLHTAM01), which can withstand temperatures of approximately 200 °C [31]. The similar base composition of these resins (both being methacrylate-based) [30,31] allowed for the extrapolation of the benefits of the full regiment of post-curing processes used in Clear Resin (FLGPCL04). High Temp Resin showed a similar untreated performance viability as Clear Resin at 45.26% ± 0.85% and was also able to be converted to be fully biocompatible (meeting the established HL-1 biocompatibility metric [54]) with the full post-print treatment regimen and with a viability of 87.74% ± 1.6%. Although this resin was significantly less biocompatible after treatments than Clear Resin, it was obvious due to the lack of mechanical damage as observed using optical microscopy that it qualitatively withstood the autoclaving process far better.

In order to assess the ability to improve this viability further, several common BioMEMS and microfluidics materials coatings that are typically used in conjunction with 3D printing of resins in microfabrication were added to the fully post-processed FormLabs High Temp Resin chips (Figure 3B). These coatings served to enhance biocompatibility: SU-8 demonstrated 88.89% ± 1.0% viability, polystyrene with 89.80% ± 1.2% viability, Medco/PET lamination with 92.31% ± 2.2% viability, gold with 93.1% ± 1.7% viability, and PDMS with 95.05% ± 0.75% viability. The polystyrene coating was found to be porous [55], perhaps causing its lower viability numbers. SU-8 was deposited in the same manner, so we suspect that it may be porous as well. The Medco/PET lamination had a tendency to be detached from the surface of the chips after extended exposure to the culture media, which could have influenced the viability obtained. Gold had surprisingly good viability, which is encouraging for exposed electrodes on a 3D-printed polymer surface. PDMS demonstrated the highest viability of all of the coatings for the FLHTAM01 resin structures, which resulted in its subsequent use as a coating for the remaining experiments.

As an additional test for increasing the viability, this resin was subjected to an extended 30-min UV treatment to assess the possibility of potentially increasing the UV post-cure time for enhanced crosslinking of dangling polymer chains. The resulting viability of 92.09% ± 0.46% (without any material coatings) suggests this is a potential course of action (result not shown in Figure 3). The FormLabs Clear Resin, when fully treated and PDMS-coated, did demonstrate a 99.42% ± 0.11% culture viability, but it still suffered from failures in the structural integrity of the material that were induced during the autoclaving cycle. As a result, the usage of High Temp Resin is preferred for 3D printed architectures.

Other 3D-printing resins may additionally be options in the development of novel BioMEMS, wearable, and microfluidics devices. In order to enhance the material palette FormLabs Dental LT (DLFLCL01) [33], FormLabs Flexible (FLFLGR0) [32], and Pro3dure GR-10 resins [34] were additionally tested after being subjected to post-treatment (IPA wash, 6 min of UV exposure, and autoclave) and material coating (Figure 3C). These resins were selected because of their commercial availability, and because they are all designated as biocompatible according to ISO standards [10,17,18,20,21,22,23,24,26,27,43,44,56,57,58,59]. Since PDMS showed the best biocompatibility results among all the coatings used with High Temp Resin, it was used as the coating in the abbreviated evaluation of these other resins. Surprisingly, the Dental LT Resin, reported as a Class IIA long-term biocompatible resin [33], demonstrated a lower assessed viability of 85.29% ± 3.4% when compared to the other two resins. Even with PDMS coating, the viability was found to be 89.66% ± 2.8%. Flexible Resin demonstrated biocompatibilities of 90.00% ± 1.7% and 93.10% ± 2.7% respectively for post-treatment and PDMS-coating, respectively. The drawback for Flexible Resin overall is its opacity, which limits its use in in vitro biological research requiring transmitted light microscopy, but this resin could find applications in wearable microfluidics, for instance [60]. Finally, the GR-10 resin demonstrated minimal biocompatibility both when fully post-treated (83.33% ± 5.2%) or coated with PDMS (85.00% ± 2.9%). The biocompatibility standard with respect to HL-1 [54] is met with all of these conditions with the exception of post-treated GR-10 structures. The reason for this result is currently being evaluated.

Additional testing was performed to demonstrate that the reported results were from the resin chips alone and material interactions with HL-1 cells, and not the result of the cells in the surrounding well area. Six fully post-treated chips of each of the resins above were adhered to a well in a 48-well plate and then carefully coated with gelatin/fibronectin solution so that only the chip was coated. Cell viability was subsequently determined using trypan blue exclusion [61]. The resulting cell viabilities, shown in Figure 3D, mirror those shown in Figure 3C.

Surprisingly, the FormLabs Clear Resin and High Temp Resin performed better overall than the commercial resins marketed as biocompatible. Obviously, nuanced differences in the composition of both resins exist; however, the high temperature resin can meet the viability threshold of 85.00% [54] while maintaining its integrity under higher heat and pressure, a necessary requirement in microfabrication. The viability of this material can be further improved with the addition of coating materials such as PDMS. This material appears to be a promising material for BioMEMS, bioelectronics, wearable, and microfluidics researchers.

**Figure 4:**
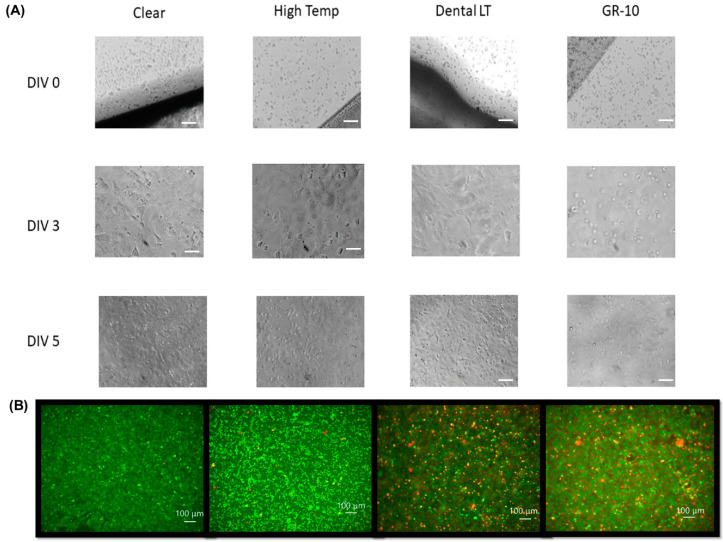
(**A**) Images of HL1 cell growth over the course of 5 days. Clear Resin, High Temp Resin, and Dental LT Resin all demonstrated expected cell growth and matting. The GR-10 resin, however, had extensive cell death with minimal growth. Confluency, or full cell coverage of the surface, is reached after DIV 5 (five days in vitro). After this time, the cells begin to overgrow and die. (**B**) Live/dead fluorescence imaging of the HL-1 cells on top of the Clear Resin, High Temp Resin, Dental LT Resin, and the GR-10 resin. The cells show excellent viability on both Clear Resin and High Temp Resin, but poor viability on Dental LT Resin and the GR-10 resin. All scale bars are 100 µm.

HL-1 cells are separated when seeded but grow to form mats (Figure 4A). These mats subsequently begin to spontaneously beat, a direct measure of healthy, active cardiomyocyte layers. This can be observed optically and can be interpreted as a direct measure of biocompatibility since the cells will not grow or beat if their viability is compromised [62]. Cells were found to spontaneously beat on all the resins except for the GR-10. Cells grown on the flexible resin were not optically observed due to the opacity of the material. Images of the cell growth and matting over the course of the five-day testing period are shown in Figure 4. Expected cell growth and matting was observed for Clear Resin, High Temp Resin, and Dental LT Resin; however, the GR-10 resin showed extensive cell death and minimal growth. Live/dead fluorescence imaging of the cells on top of the Clear Resin, High Temp Resin, Dental LT Resin, and the GR-10 resin was performed using a Keyence BZ-X800 Confocal Microscope (Figure 4B). Cells on top of the Clear Resin and High Temp Resin showed excellent viability, while those on top of the Dental LT Resin and the GR-10 resin showed less desirable viability. This is expected from the results presented above.

## 4. Conclusions

The expansion of 3D printing into the microfluidic and BioMEMS fields has been suppressed by the lack of transparency in the chemical compositions of commercially available resins, as well as the minimal amount of biocompatibility data currently available in literature. We have taken what we believe to be the first steps towards establishing biocompatibility of various resins with respect to electrogenic cells, utilizing both post-processing treatments and material coatings. Specifically, the results presented here provide a clear pathway toward applying these resins toward 3D printing-based 2D and 3D microelectrode array (MEA) devices [35,55,63].

Here, we demonstrated how each post-process treatment step improved the biocompatibility of methacrylate-based commercial resins, as well as how this biocompatibility is further addressed through material coatings. To achieve the viability threshold for HL-1 cells, any combination of our chosen post-process treatments (IPA, UV, thermal, and autoclaving) resulted in viable cultures (well above the 85.00% threshold) with FormLabs Clear Resin. With respect to our chosen coatings (PDMS, PS, SU-8, Au, Medco/PET) on this resin, we observed similar performances of above the 85.00% threshold using every coating, with PDMS outperforming the others. Comparing the various resins (i.e., Formlabs Clear Resin, Flexible Resin, High Temp Resin, Dental LT Resin, Pro3dure’s GR-10), all with the full set of combined post-process treatments, additionally met or exceeded the 85.00% threshold, with the exception of the GR-10 resin. Utilizing the best performing coating (PDMS), we were able to improve all treated resin biocompatibilities, including bringing the GR-10 resin up to meet the 85.00% viability threshold.

The compositional cues from these resin materials and their relationships to the observed biocompatibility are being explored currently by our group as well as other researchers. The preliminary results reported in this work are a dramatic step forward in the assessment of the biocompatibility of 3D-printed photocurable resins with respect to electrogenic cells and provides a pathway for the potential development of fully functional, 3D printing-based, in vitro BioMEMS, bioelectronic, wearable, and microfluidic devices.
